# Chikungunya resurgence in Italy: a signal of vectorborne viruses changing pattern in the era of climate change?

**DOI:** 10.3389/fpubh.2026.1787941

**Published:** 2026-03-19

**Authors:** Rita Cuciniello, Carlo Signorelli, Giovanni Rezza

**Affiliations:** School of Medicine, Vita-Salute San Raffaele University, Milan, Italy

**Keywords:** *Aedes albopictus*, chikungunya, chikungunya virus, One Health, Wolbachia

Chikungunya virus infection is a growing public health concern because of its high attack rate and potential for long-term disability. Beyond the acute febrile illness, a substantial proportion of infected individuals develop chronic, often debilitating manifestations, in particular persistent inflammatory arthralgia and neuropathic pain, which may persist for months or even years and significantly impair quality of life (1).

Recent evidence has also highlighted the strong inflammatory potential of chikungunya infection, particularly relevant for older adults and individuals with pre-existing cardiovascular conditions, in whom systemic inflammation may exacerbate underlying disease and increase the risk of complications (2, 3). Therefore, Chikungunya outbreaks are associated not only with a short-term healthcare burden but also with prolonged individual disability and sustained pressure on health systems (1).

In this context, the occurrence of chikungunya outbreaks outside traditionally endemic areas raises specific concerns for public health preparedness and response.

The first outbreak of chikungunya in a temperate climate country was identified in northern Italy in 2007 (4). Ten years later, chikungunya reappeared in a different area of Italy, not far from Rome (5), causing a secondary outbreak in the south (6). Apart from these major outbreaks, sporadic cases or small clusters have been reported over the years in the western Mediterranean area (7).

This year, 734 cases of chikungunya in 75 different clusters have been identified in southern France since late spring, partly related to epidemic waves involving overseas French islands in the Indian Ocean (8). The largest cluster, with 141 cases, has been reported in Antibes. However, far from the Mediterranean coast, 103 cases have been reported in Bergerac. These data are reported in the ECDC Seasonal Surveillance for Chikungunya Virus Disease in the EU/EEA for 2025.

In July and August, a total of 384 chikungunya cases were detected in several areas of northern Italy: two major outbreaks occurred in the Emilia-Romagna region (318 cases in Carpi and other sites in Modena province) and Veneto (61 cases in Verona province), while the remaining few cases were reported again in Emilia (two cases in Bologna, one in Piacenza and one Rimini), and in Tuscany (one case) (9, 10).

**Figure 1:**
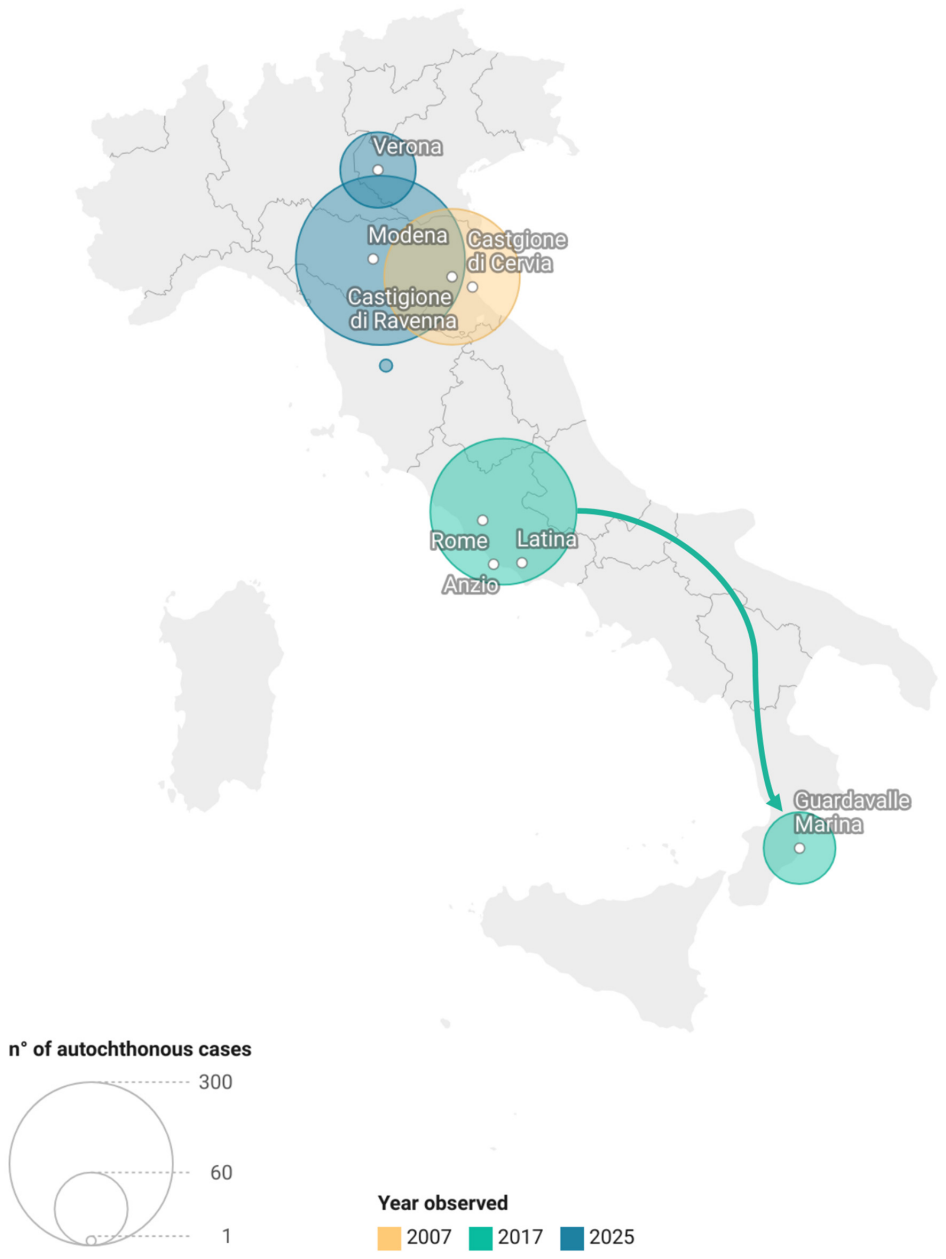
Geographic distribution and number of autochthonous chikungunya cases reported in Italy, by year of observation (2007, 2017 and 2025).

As shown in Figure 1, the 2025 epidemiological feature, with a concentration of outbreaks in northern Italy, shares similarities but also differences when compared with the patterns observed in 2007 and 2017. The 2007 outbreak was characterized by a single large, localized epidemic in northern Italy (205 cases), occurring in a setting of limited preparedness and low awareness for autochthonous transmission in temperate regions (4). In the year 2017, a chikungunya outbreak in central Italy was followed by asecondary autochthonous outbreak in southern Italy: transmission chains were initially detected in the Lazio Region (297 cases), with a few cases observed also in the city of Rome, and subsequently in Calabria (132 cases); epidemiological investigations supported a correlation between the two events (5, 6, 11). On the other hand, in 2025, independent multiple outbreaks and clusters occurred in northern Italy, particularly in Emilia-Romagna and Veneto, showing a fragmented spatial distribution (8).

Repeated introductions of the virus from endemic areas due to increased human mobility, and permissive environmental and climatic conditions, may have contributed to the reemergence of chikungunya in Italy. Usually, chikungunya outbreaks in the Mediterranean area are the result of virus introduction from tropical countries characterized by high levels of virus circulation. Synchronicity between the occurrence of such epidemics in tropical countries which are touristic destinations or are involved in relevant migration patterns and the hot season in Europe plays a major role (4, 5). This was particularly clear during the last summer, when a large number of cases were reported in mainland France due to multiple introductions from Indian Ocean overseas French islands (8). Then, as highlighted by mathematical models, local transmission of tropical vectorborne viruses such as chikungunya, as well as dengue, is mainly driven by case importation in areas where environmental and climate conditions are permissive (12). This may explain why most autochthonous cases of chikungunya occurred in northern and central Italy, where population mobility due to both international tourism and regular migration patterns via air transport has a major impact compared to the south of the Country. To this purpose, it should be mentioned that in the last couple of years several dengue outbreaks were reported in Italy, as a consequence of large epidemic waves of dengue in touristic destinations (4).

In 2025, Italy hosted the Jubilee, a prolonged mass gathering event associated with increased international mobility and sustained population influx. In the Lazio region, preventive vector surveillance and control measures were implemented in advance as part of preparedness activities (13). Autochthonous chikungunya cases were not reported in and around the city of Rome, while high West Nile virus activity was documented in the Region (14). This observation suggests that, while preventive vector control measures are essential to reduce the risk of mosquito-borne diseases transmitted by Aedes mosquitoes, they are not sufficient *per se* to prevent arboviral circulation, which is strongly influenced by virus-specific ecological and epidemiological factors, including patterns of introduction and reservoir dynamics. In the case of chikungunya, the lack of local transmission during the Jubilee may also reflect an absence of effective virus introduction.

Finally, climatic variables may influence chikungunya transmission through biological mechanisms that affect both vector biology and viral replication. Elevated temperatures accelerate the mosquito developmental cycle, increase biting frequency, and shorten the extrinsic incubation period of the virus, thereby enhancing transmission efficiency (15, 16). In addition, alternating heat waves and intense precipitation can increase the availability of breeding sites, particularly in urban and peri-urban settings, sustaining higher vector densities (17). Climate change may also increase the risk of larger outbreaks and even virus overwintering in case of a prolonged hot period. In fact, combined with early virus introduction, prolonged mosquito activity may give the virus more time to spread. Moreover, milder winters could favor mosquito reproduction and activity all year. To this regard, dengue virus overwintering was hypothesized having occurred in 1927–28 in Athens, where a dengue outbreak sustained by *Aedes aegypti* (which disappeared from the Eastern Mediterranean coasts after 1930s) occurred over two successive years (18). Whether climate change may have played a role in the current outbreaks remains unclear. Italy has always been a part-time tropical Country, and *Aedes albopictus*, which was introduced in the early ‘90s, is largely established in almost all the national territory (19). However, the increasing frequency of extreme weather events, such as heat waves alternating with intense precipitation, may amplify transmission potential in already permissive settings.

Since it has been estimated that the reproduction number (Rt) tends to decline shortly after outbreak detection and consequent vector control measures implementation, early detection of local transmission chains is important. This can be achieved through strengthened surveillance systems, including enhanced human case identification, timely laboratory confirmation, and increased clinical suspicion among health-care workers for autochthonous cases, even in the absence of travel history. In addition, raising awareness among the general population about early symptoms and the possibility of local transmission may help reduce delays in diagnosis and response (12).

However, in the context of a changing epidemiological pattern of Aedes-borne viruses in permissive areas, priority should be given to early, regular, and widespread preventive measures aimed at reducing vector density. These include systematic control of mosquito breeding sites through larvicidal treatments in both public and private settings, removal of water collections, and strengthened risk communication to the population and health-care providers (20). While the availability of new vaccines may represent a potential complementary tool in selected scenarios, their role in outbreak settings remains to be clearly defined, given current limitations related to time to immunity and age eligibility. Recent recommendations from the Italian Society of Travel and Migration Medicine for the use of the chikungunya vaccine support an approach aimed at reducing the importation of cases among residents traveling abroad for tourism or work (21). These recommendations transform Chikungunya prevention from an emergency management approach to a structured defense, capable of meeting both the needs of international travelers and protecting the community from autochthonous outbreaks, confirming Italy's commitment to combating viral threats that can no longer be considered distant.

Innovative biological approaches targeting the vector itself could further strengthen prevention strategies, particularly when integrated with early, regular and widespread control of vector breeding sites. The use of *Aedes* mosquitoes infected with Wolbachia, an intracellular bacterium capable of reducing vector competence for several arboviruses, including chikungunya, has emerged as a promising complementary intervention (22). While vaccination primarily protects the human host reactively during outbreaks, Wolbachia acts upstream at the root of transmission, potentially enhancing the overall resilience of public health systems. From a climate-adaptation perspective, this strategy might provide a persistent biological barrier to transmission in settings where milder winters and prolonged mosquito activity favor viral survival. Evidence from large-scale field studies conducted in countries such as Brazil and Australia supports the effectiveness of this approach, although challenges related to scalability, community acceptance, and regulatory frameworks should be acknowledged (23, 24).

In conclusion, the increased number and extent of *Aedes albopictus* mosquito-borne virus outbreaks in the Mediterranean area and changing climate conditions pose a new challenge to public health. To contain and/or mitigate emerging epidemic events, preparedness and response activities should be strengthened, adopting a holistic approach consistent with the “One Health” framework.
